# Plant Species of Sub-Family Valerianaceae—A Review on Its Effect on the Central Nervous System

**DOI:** 10.3390/plants10050846

**Published:** 2021-04-22

**Authors:** Gitishree Das, Han-Seung Shin, Rosa Tundis, Sandra Gonçalves, Ourlad Alzeus G. Tantengco, Maria G. Campos, Rosaria Acquaviva, Giuseppe Antonio Malfa, Anabela Romano, Joyce Ann H. Robles, Mariel Q. Clores, Jayanta-Kumar Patra

**Affiliations:** 1 Research Institute of Biotechnology & Medical Converged Science, Dongguk University-Seoul, Goyang-si 10326, Korea; gdas@dongguk.edu; 2 Department of Food Science & Biotechnology, Dongguk University-Seoul, Goyang-si 10326, Korea; spartan@dongguk.edu; 3 Department of Pharmacy, Health and Nutritional Sciences, University of Calabria, 87036 Cosenza, Italy; rosa.tundis@unical.it; 4 MED—Mediterranean Institute for Agriculture, Environment and Development, Universidade do Algarve, Faculdade de Ciências e Tecnologia, Campus de Gambelas, Ed. 8, 8005-139 Faro, Portugal; smgoncalves@ualg.pt (S.G.); aromano@ualg.pt (A.R.); 5 College of Medicine, University of the Philippines Manila, Manila 1000, Philippines; ogtantengco@up.edu.ph (O.A.G.T.); jhrobles@up.edu.ph (J.A.H.R.); 6 Observatory of Drug-Herb Interactions, Faculty of Pharmacy, University of Coimbra, Heath Sciences Campus, Azinhaga de Santa Comba, 3000-548 Coimbra, Portugal; mgcampos@ff.uc.pt; 7 Coimbra Chemistry Centre (CQC, FCT Unit 313) (FCTUC), University Coimbra, Rua Larga, 3004-535 Coimbra, Portugal; 8 Department of Drug and Health Science—Biochemistry Section, University of Catania, Viale A. Doria 6, 95125 Catania, Italy; racquavi@unict.it (R.A.); g.malfa@unict.it (G.A.M.); 9 Department of Chemistry, University of Connecticut, Storrs, CT 06269, USA; mariel.clores@uconn.edu

**Keywords:** Valerianaceae, anxiolytic, sedative, myorelaxant, antidepressant, biological activities, clinical studies, phytochemicals, valerian

## Abstract

Valerianaceae, the sub-family of Caprifoliaceae, contains more than 300 species of annual and perennial herbs, worldwide distributed. Several species are used for their biological properties while some are used as food. Species from the genus *Valeriana* have been used for their antispasmodic, relaxing, and sedative properties, which have been mainly attributed to the presence of valepotriates, borneol derivatives, and isovalerenic acid. Among this genus, the most common and employed species is *Valeriana* *officinalis*. Although valerian has been traditionally used as a mild sedative, research results are still controversial regarding the role of the different active compounds, the herbal preparations, and the dosage used. The present review is designed to summarize and critically describe the current knowledge on the different plant species belonging to Valerianaceae, their phytochemicals, their uses in the treatment of different diseases with particular emphasis on the effects on the central nervous system. The available information on this sub-family was collected from scientific databases up until year 2020. The following electronic databases were used: PubMed, Scopus, Sci Finder, Web of Science, Science Direct, NCBI, and Google Scholar. The search terms used for this review included Valerianaceae, *Valeriana*, *Centranthus*, *Fedia*, *Patrinia*, *Nardostachys*, *Plectritis*, and *Valerianella*, phytochemical composition, in vivo studies, Central Nervous System, neuroprotective, antidepressant, antinociceptive, anxiolytic, anxiety, preclinical and clinical studies.

## 1. Introduction

Valerianaceae, the sub-family of the family Caprifoliaceae (order Dipsacales), contains about 315 species of annual and perennial herbs, distributed, throughout the world, except Australia and New Zealand, usually at high elevations. Achene fruits, absence of endosperm, bilaterally symmetric or sporadically asymmetric and sympetalous flowers, three-carpellate and inferior ovaries, one fertile carpel at maturity, and an anatropous ovule are the main characteristics of Valerianaceae sub-family species [1]. Valerianaceae sub-family showed significant diversity in flowers and fruits morphology. Probably, the most remarkable variation in the morphology of flowers is related to the stamens number ranging from 4 to 1. Donoghue et al. [2] described a reduction from the ancestral condition of 4 to 3 stamens in the Valerianaceae core, followed by an additional reduction to two stamens in *Fedia* genus and one stamen in *Centranthus* genus.

The calyx can be completely lacking as in some *Valeriana* species, reduced to small teeth as in *Fedia* and *Valerianella*, leafy and persistent as in *Nardostachys*, or pappus-like and featherly as in some *Valeriana* species and in *Centranthus*. The genera of this sub-family include *Valeriana* L., *Centranthus* Lam. and DC., *Fedia* Gaertn., *Patrinia* Juss., *Nardostachys* DC., *Plectritis* (Lindl.) DC., and *Valerianella* Mill. Phytochemical studies revealed the presence of sesquiterpenes, valepotriates, alkaloids, flavonoids, organic acids, and their derivatives, as characteristic classes of constituents in Valerianaceae plants [3,4,5]. It is generally accepted that valepotriates are common in Valerianaceae species and are responsible for their sedative properties. The present review is designed to report the current knowledge on the plant species that belong to the sub-family Valerianaceae, their phytochemicals, and their uses in the treatment of several diseases with particular emphasis to the action on the central nervous system (CNS). All collected data have been obtained from different databases such as PubMed, Scopus, Sci Finder, Web of Science, Science Direct, NCBI, and Google Scholar.

## 2. Habitat, Distribution, and Traditional Uses of Valerianaceae Sub-Family

Valerianaceae species are mainly found around the Northern Hemisphere. Overall, their distribution matches that of other Dipsacales clades. Even though the Asiatic origin, actually the center of diversity Valerianaceae sub-family is located in South America, where several morphological forms, from rosette plants to annual vine-like species to microphyllous shrubs, are found in different habitats [2,6,7,8]. Several species of the genus *Valeriana* are abundant in the Andes. Peru is the richest country, but there is a high number of species also in Ecuador, Chile, Colombia, and Argentina [9]. Species present in northwestern Argentina, and northern Chile are generally inhabitants of the arid zone and are taxonomically related to species found in the northern Andes [9]. *Patrinia* species are principally distributed in China, Korea, Japan, and Siberia. *Nardostachys grandiflora* DC. (syn. *Nardostachys chinensis* and *Nardostachys jatamansi*) and *Nardostachys scrophulariiflora* (syn. *Picrorhiza scrophulariiflora*) are alpine perennial herbs found only in the Himalayas [10].

Numerous Valerianaceae species have a traditional use. Several species are used for their biological properties while some species are used as food. Species from the genus *Valeriana* have been used for their antispasmodic, relaxing, and sedative properties that have been mainly attributed to the presence of valepotriates, borneol derivatives, and isovalerenic acid [11]. Among this genus, the most common and employed species is *Valeriana officinalis* L. Its roots have long been traditionally used for their sleep-promoting, anxiolytic, sedative, and antispasmodic activities [12,13,14]. In Brazil, it has been used for its hypnotic, anticonvulsant, and anxiolytic properties [15]. In Europe, *V. officinalis* is used for treating anxiety and restlessness; in the United States, it is mainly employed for its sleep-promoting activity [12].

*Patrinia* species are commonly used in Asian medicine to treat peri-appendicular abscesses, dysentery, erysipelas, conjunctival congestion, lung carbuncle, post-partum disease, and leucorrhea [16]. The young leaves and shoots of *Centranthus ruber* (L.) DC. (*Valeriana rubra* L., commonly known as red valerian, red spur valerian, and spur valerian) are used in Italy as depurative [17]. *N. chinensis* has been used in some traditional medicines, including Chinese, Korean, and Ayurvedic medicine to treat epilepsy, hysteria, hyperglycemia, dyslipidemia, headache, stress indigestion, heart palpitations, mental weakness, cholera, and leprosy [18]. The leaves of *Fedia cornucopieae* (L.) Gaertn. are eaten in southern Italy (Sicily) raw in salads or cooked (browned in oil, fried, in omelets, and meatballs) [19].

## 3. Effect on the Central Nervous System

Among Valerianaceae plants, species from the genus *Valeriana* (e.g., *V. officinalis* L., *V. jatamansi* Jones, *V. fauriei* Briq., *V. amurensis* Smir. ex Kom, *V. glechomifolia* Meyer, *V. polystachya* Smith, etc.) are the most used to treat CNS-related disorders (Table 1). In the last decades, many studies have validated the traditional uses of these species. *V. officinalis* is the most used particularly for its sedative, anticonvulsant, tranquilizing, and anxiolytic properties. According to the European Medicine Agency, extracts from this species roots can be used to alleviate mild nervous anxiety and sleep disorders [20]. *V. officinalis* is available as an herbal supplement and is extensively utilized to cure anxiety disorders. Its anxiolytic effects have been studied by different authors using different models. Hattesohl et al. [21] investigated in vivo the myorelaxant, anxiolytic, sedative, and antidepressant properties of different extracts from this species. None of the studied extracts showed myorelaxant or sedative activity up to limit doses of 500 or 1000 mg/kg b.w. Nevertheless, some extracts showed pronounced anxiolytic activity in the elevated plus-maze assay and one extract, after subacute treatment, showed anti-depressant activity in the forced swimming assay, allowing to conclude that the anti-depressant and anxiolytic effects may contribute to the positive effects of valerian to improve sleep quality.

The effect of oral administration of *V. officinalis* root extract on physical and psychological stress response was investigated in mice by using a communication box [22]. Obtained data suggested that this extract could suppress stress responses through the modulation of the changes in the turnover of norepinephrine and serotonin in the amygdala and hippocampus, and through the control of corticosterone plasma levels. 

In a study by Murphy et al. [23] *V. officinalis* root extract was intraperitoneally administered to rats and the number of entries and time spent on the open arms of an elevated plus-maze was evaluated. A reduced anxious behavior was observed in extract-treated rats in comparison to the control group and valerenic acid was suggested as the main anxiolytic component in the extract. However, other studies indicate that other constituents, namely borneol, lignans, and flavonoids, also exhibited anxiolytic and sedative activity [24,25,26]. In addition, studies with other *Valeriana* species containing low amounts of valerenic acid (e.g., *V. edulis*) showed similar properties [27].

The anticonvulsant effects of various extracts from *V. officinalis* were investigated in mice using the temporal lobe epilepsy model showing that administration of aqueous extract significantly decreased seizure activity in amygdala-kindled rats [63]. The anticonvulsant effect of an aqueous extract against clonic seizure threshold induced by pentylenetetrazole in mice [48]. Using a different mode, adult zebrafish [51], observed that ethanol extract noticeably improved the anticonvulsant effect of the anti-epileptic drugs clonazepam and phenytoin thus benefiting epilepsy treatment.

Derived Neurotrophic Factor (BDNF) has a crucial role in the CNS. The levels of BDNF must be maintained at an adequate concentration to allow neurotransmission to occur at an ideal level and prevent several mental diseases. A significant increase in the BDNF expression compared to control in SH-SY5Y cell lines treated with *V. officinalis* extract was observed allowing us to conclude that the antidepressant effects are mostly due to valerenic acid [64,65].

Yoo et al. [52] investigated the neuroprotective action from *V. officinalis* root extracts in gerbils after transient cerebral ischemia. The results revealed that the oral pretreatment with 100 mg/kg of extract decreased microglial activation and lipid peroxidation protection against ischemic damage in the hippocampal pyramidal neurons. In another study, the same root extract (100 mg/kg) and valerenic acid (340 μg/kg) was orally administered to control mice and aged mice (previously treated with D-galactose) to evaluate their effects on cell proliferation, memory function, and neuroblast differentiation in the mouse dentate gyrus [49]. Both enhanced the preferential exploration of new objects (novel object recognition test) and the escape latency, platform crossings, swimming speeds, and spatial preference for the target quadrant (Morris water maze test). They also improved cognitive function, promoted cell proliferation, neuroblast differentiation, and decreased the plasma corticosterone levels in aged mice.

The neuroprotective properties of *V. officinalis* extract and its therapeutic potential for neurological disorders have been reported. The extract showed protective effects against toxicity induced by rotenone in *Drosophila melanogaster* [66]. Rotenone is a chemical substance widely used as a pesticide that acts within the respiratory chain causing oxidative damage. Animals’ treatment with this substance has been used as a model to study brain disorders linked with redox imbalance. Amaral de Brito et al. [56] recently observed that *V. officinalis* extract has protective effects against in vitro cytotoxicity induced by rotenone in rat glioma C6 cells, by a novel action on the cortical spreading depression.

The antinociceptive effects of a *V. officinalis* hydroalcoholic extract were demonstrated in adult male Balb/c mice [50]. When mice were injected intraperitoneally at the maximum dose tested (800 mg/kg), the somatic pain was successfully reduced, the antinociceptive activity was clearly expressed, and the number of abdominal writhings significantly decreased.

Acetylcholinesterase (AchE) inhibitors have been widely recognized as an effective treatment for Alzheimer’s disease (AD) and AChE inhibitory activity of compounds from *V. officinalis* (sesquiterpenes and a monoterpenoid) have been also validated in mice both in vitro and in vivo [53].

*V. jatamansi* (synonymous *V. wallichii* DC.), popularly named as Indian valerian, is considered as an Asian counterpart for the European *V. officinalis*, is another important medicinal plant from the genus *Valeriana,* broadly recognized for its uses in anxiety, insomnia, epilepsy, and hysteria treatment (Nadkarni, 2001). Many studies are reporting the pharmacological properties of this species including the sedative, tranquilizing and neuroprotective effect, and its capacity to attenuate stress, anxiety, and depression (Table 1) [67]. The anti-anxiety effect of its ethanol extract was studied on empty bottle-stimulated rats using the open field and the elevated plus-maze tests. Four bioactive compounds (hesperidin, isochlorogenic acid A, isochlorogenic acid B, and isochlorogenic acid C) were identified as the chemical markers for that effect [46]. There are some reports about the antidepressant effect of extracts and essential oils from this species [60,68,69,70]. The nitric oxide pathway is involved in the antidepressant properties of essential oil from a chemotype of this species in mice [60]. The essential oil components inhibit nitric oxide to a critical concentration changing the vesicular release of norepinephrine and serotonin, two neurotransmitters implicated in depression.

An aqueous roots extract of *V. jatamansi* was also investigated for its effects on the sleep pattern of Sprague-Dawley rats by analyzing sleep-wake profile, electroencephalogram delta action during sleep, and estimating regional brain monoamines [61]. The extract ameliorated sleep disturbances and improved sleep quality, which can be related to the modulation of regional brain monoamines.

Several reports are describing the neuroprotective effects of extracts and compounds from *V. jatamansi* [43,62]. The neuroprotective effects of bakkenolides and iridoids against 1-methyl-4-phenylpyridinium (MPP+)-induced neuronal cell death in human dopaminergic neuroblastoma SH-SY5Y cells were demonstrated [43,44]. 1-Metil-4-fenil-1,2,3,6-tetraidropiridine (MPTP), a precursor of the neurotoxin MPP+, is widely used to study PD insult. The neuroprotective effects against MPTP in mice by mitigating oxidative stress and inflammatory damage were also reported [62].

*V. fauriei* is also described as an antidepressant [37], vasorelaxant [71], and anxiolytic [72]. Yoon et al. [34] demonstrated its antinociceptive effects in a fibromyalgia animal model provoked by intermittent cold stress. Its effects in reducing the occurrence of prenatal depression and schizophrenia in rats were also reported [38].

*V. amurensis* (roots and rhizomes) has been extensively employed in Chinese medicine for the treatment of neurological disorders. The petroleum ether extract improves sleep by reducing the sleep latency and delaying the sleep duration of mice [73]. Using human cell lines and mice models Wu et al. [36] recently reported the anti-inflammatory and neuroprotective properties of kissoone B and extracts of this species, suggesting its capacity for neurological diseases treatment.

*Nardostachys jatamansi* (D. Don) DC. (synonymous *Nardostachys chinensis* Batalin) belongs to a different Valerianaceae genus and has also been extensively reported as affecting CNS disorders (Table 1). The anti-stress properties of this species have been investigated by several authors and using different animal models. Lyle et al. [74] using a rat model of chronic fatigue syndrome, observed a significant reversion of the locomotor activity and the anxiety state in stressed animals, after the oral administration of the extract (200 and 500 mg/kg b.w.). The extract showed the capacity to manage lipid peroxidation, nitrite, superoxide dismutase, and catalase activities indicating that the antioxidant properties of this extract can be responsible for its anti-stress effects. Later, Lyle et al. [30] used a cold restraint stress model to investigate the biochemical and neurochemical alterations induced by oral administration of a hydro-ethanolic extract in the same doses and observed alterations on rats enzymatic and non-enzymatic antioxidant system, and alleviated oxidative stress and neurochemical alterations induced by cold restraint stress.

This species has been traditionally used as a tranquilizer and sedative agent and many investigations demonstrating its anxiolytic and sedative properties. The sedative effect of extracts and isolated compounds applied by inhalation were previously reported [28,75,76]. Takemoto et al. [76] investigated the sedative effects of oxygenated compounds applied by inhalation in an excitatory mouse model treated with caffeine and showed that aristolen-1(10)-en-9-ol exerted its effects via the GABAergic system. The anxiolytic effects of *N. jatamansi* extract were investigated after oral administration to mice (250 mg/kg) by using different behavioral tests, such as elevated plus maze, light-dark box test, open field test, and Vogel’s conflict test [32]. A 7 days treatment with the extract showed significant anxiolytic effects and increased levels of brain monoamine and GABA neurotransmitter, suggesting that anxiolytic effects are mediated by activating the GABAergic receptor complex.

Recently, the potential of some *Nardostachys* species to alleviate AD-like symptoms in *Caenorhabditis elegans* AD pathological model was investigated and some compounds can delay AD worm paralysis [29,77].

Some reports are describing the serotonin transporter regulating the activity of compounds from *Nardostachys* species, like sesquiterpenoids and nardonaphthalenones [78,79]. Serotonin transporter is responsible for the reuptake of 5-hydroxytryptamine into presynaptic neurons and plays an important role in the pathophysiology of neuropsychiatric conditions, like depression, anxiety, or obsessive-compulsive disorder.

Many investigations have been performed in animals to assess the anticonvulsant properties of *N. chinensis*. An ethanol extract significantly increased the seizure threshold in a dose and time-dependent manner in a rats’ model of electric shock-induced seizures [80]. The anticonvulsant effect of *N. jatamansi* extract (400 mg/kg b.w.) in a maximal electric shock model was less than that of the standard sodium valproate (300 mg/kg b.w.) [81]. The neuroprotective effect of an ethanol extract from the roots of this species against Aβ toxicity was studied in vitro using a cell culture system and in vivo using a *Drosophila* AD model [82] and the results suggest that the neuroprotective effect observed can be linked with the extract antioxidant and anti-inflammatory properties and its inhibitory effect against extracellular-signal-regulated kinase signaling. Indeed, several extracts, fractions, and compounds from this species exhibited anti-neuro inflammatory by different mechanisms [31,32,33,34,83,84].

## 4. Molecular Mechanism of Action of Pharmacological Potential

*V. officinalis* extracts are one of the most popular herbal medications used to alleviate insomnia, anxiety, epilepsy, and other conditions of the CNS. Several studies have been conducted to clarify the mechanisms implicated in the pharmacological effects of these extracts. γ-Aminobutyric acid (GABA) is the main inhibitory neurotransmitter in the CNS. GABA is crucial for normal brain function and a reduction in its concentration has been associated with several neurological conditions as is the case of epilepsy, AD, Parkinson’s disease, Huntington’s disease, etc. If GABA balance is perturbed, conditions like depression, sedation, anxiety, restlessness, and insomnia may also arise [85]. Due to the blood–brain barrier, GABA cannot be easily introduced into the CNS, thus the inhibition of GABA aminotransferase, the enzyme responsible for its degradation, has been the target for the adjustment of GABA amounts in the CNS. The mode of action of valerian compounds is associated with the modulation of GABAergic transmission via inhibition of GABA transaminase, interaction with GABA receptor/benzodiazepine, and intervention in uptake and intake of GABA in synaptosomes.

Studies with animal showed that *V. officinalis* potentiates the GABAergic neuronal transmission in the brain via the improvement of GABA release and inhibition of the degradation of GABA via the inhibition of GABA-transaminase [86,87,88]. A recent study of molecular docking and molecular dynamics simulation suggests that valerian compounds could be valuable resources for the development of GABA-transaminase inhibitors [55]. Valle-Mojica et al. [89] suggested an interaction with glutamatergic receptors as a possible mechanism by which valerian exhibit its action in the central nervous system. Valerian compounds like valerenic acid and valerenol also displayed allosteric modulation of GABA receptors [90,91]. In vitro studies indicated that both the activation of adenosine A_1_ receptor and GABA_A_ receptors separately contribute to the valerian capacity to induce sleep [92]. It was also observed that factors like solvent extraction and the stability of the extract affect the selectivity for the interactions for glutamate receptors [93].

Recently Li et al. [47] demonstrated that the antidepressant activity of the iridoid-rich fraction from *V. jatamansi* in a chronic unpredictable mild stress mouse model is exerted by regulating several metabolic pathways such as the synthesis of neurotransmitters, the tricarboxylic acid cycle, and amino acid metabolism. Recent studies also using the same model indicated that that gut flora structures and regulation of serotonin, norepinephrine, substance P, and corticotropin-releasing factor in the brain and intestine, can be implicated in the antidepressant properties of this plant [94]. The enhancement of noradrenergic and/or dopaminergic transmission induced by valepotriates from this species in the mouse brain can be related to the facilitation of GABA [59].

Studies from Shahidi et al. [50] indicated for the first time that the antinociceptive effects of *V. officinalis* are mediated by the serotonergic and dopaminergic systems. Monoamine neurotransmitters in the central nervous system, mostly serotonin (5-hydroxytryptamine, 5-HT) and norepinephrine, are crucial in regulating cognition, emotion, and mood. These neurotransmitters also play an essential role in the stress response and the mechanism of antidepressant action. It was observed that *V. officinalis* extract can suppress physical and psychological stress responses by controlling the variations in these neurotransmitters turnover in the hippocampus and amygdala [22], and reduces the plasma corticosterone levels in D-galactose-induced aging mice [49]. Dichloromethane extracts from *V. wallichii* significantly improve norepinephrine and dopamine amounts with no changes in serotonin amounts [59]. The effects of *V. adscendens* extracts on the central nervous system have been associated with the interaction with serotoninergic, dopaminergic, and noradrenergic receptors [95]. On the other hand, studies from Lee et al. [39] suggest that the antinociceptive effects of *V. fauriei* can be related to modulatory effects on the BDNF signaling pathway in the hippocampus and medial prefrontal cortex. Recently, Choi et al. [96] demonstrated that this species exerts antidepressant effects through its anti-inflammatory and antioxidant effects by inhibiting BDNF expression.

Valepotriates from *V. jatamansi* inhibit Ca_v_2.2 and Ca_v_3.1 calcium channels in a selective way which is coherent with the analgesic action of this species in relieving gastrointestinal and rheumatic pain [97]. Moreover, studies by Dong et al. [98] indicated that its use for alleviating abdominal distention and pain may be mediated through Ca_v_2.2 channel. Although the information about the anticonvulsant effects of valerian is scarce, studies from Rezvani et al. [63] suggested that it is mediated through the activation of the adenosine system.

Santos et al. [54] demonstrated that different compounds from *V. officinalis* extract may trigger distinct mechanisms involved in neuronal cell protection in PD. Hesperidin, and probably linarin, alleviate oxidative stress effects during ATP depletion due to its capability to binding SUR1. On the other hand, valerenic acid and apigenin avoid cortical hyperexcitation by stimulating neuronal cells from the substantia nigra to release GABA on the brain stem. *V. amurensis* showed protective effects on amyloid-beta (Aβ)-induced toxicity in PC12 cells [99] and capacity to improve Aβ-induced cognitive dysfunction in mice by two mechanisms, by enhancing acetylcholine and choline acetyltransferase activity and thus improving cerebral cholinergic function, and by protecting neurons from Aβ-induced apoptosis [35].

The anti-neuroinflammatory effects of *N. jatamansi* extracts and isolated compounds have been described by different authors. Ko et al. [83] reported that four nardosinone-type sesquiterpenes showed anti-neuroinflammatory action on lipopolysaccharide (LPS)-induced immortalized murine brain microglia BV2 cell lines by inhibiting NF-κB- and MAPK-mediated inflammatory pathways. The anti-neuroinflammatory effects of two sesquiterpenoids from this species, desoxo-narchinol A and narchinol B, in BV2 and the primary microglial cell, was also reported, which is related to the inhibition of LPS-induced expression of iNOS and COX-2 enzymes the suppression of pro-inflammatory cytokines (IL-1b, IL-6, and TNF-a). Yoon et al. [33] showed that the inhibition of the NF-κB signaling pathway was also implicated in the anti-neuroinflammatory effect of three sesquiterpenoids from this species. Recently, Kim et al. [84] reported the anti-neuroinflammatory effect of desoxo-narchinol A and narchinol B in microglial cells by up-regulating of nuclear transcription factor erythroid-2-related factor 2/heme oxygenase-1 signaling. The mechanisms of action of Valerianaceae plants have not been fully investigated in humans. The effects of *V. officinalis* extract on cortical excitability were evaluated in humans with transcranial magnetic stimulation [100] and it was observed that a single oral dose adjusts intracortical facilitatory circuits.

## 5. Other Pharmacological Potential of Valerianaceae

As aforementioned some Valerianaceae species, particularly from the *Valeriana* genus, are well investigated for their pharmacological potential on the central nervous, namely anxiolytic, antidepressive, antinociceptive, and anticonvulsant, etc. However, various *Valeriana* species are still understudied for other biological properties. Among all the species, *V. officinalis*, *V. jatamansi* (syn. *Valeriana wallichii*), *V. hardwickii* Wall, and *V. stenoptera* Diels. are the ones that show better potential for further investigation in drug discovery. The bioactivity of root extracts from *V. officinalis* is mainly associated with its anxiolytic compounds, as the valerenic acid and its biosynthetic precursors, valerenal and valerenadiene. β-Caryophyllene is another sesquiterpenoid present in the extracts but is more associated with the anti-inflammatory effect, and in this case, *V. officinalis* and *V. wallichii* still need to be more deeply studied. Regarding anti-viral, hepatoprotective, or immune stimulant activities, no relevant data was found in the last ten years. The most recent research with *Valeriana* spp. is published in anti-anxiety, anti-bacterial, anti-cancer, anti-depressive, and cardiovascular effects, and they are summarized below for a better understanding of the data available.

### 5.1. Antibacterial Effect

Regarding the antibacterial activity of *Valeriana* spp. extracts few data are available [101]. The aerial parts of *V. wallichii* DC (Valerianaceae) were evaluated by Khuda et al. [101], for their antifungal and antibacterial activities. The authors prepared two extracts with the aerial parts of the plant, one with chloroform and the other with hexane, and both showed significant bioactivity [101]. It was also reported antibacterial activity for *Staphylococcus aureus* and *Pseudomonas aeruginosa* by four sesquiterpenoids isolated from the roots of *V. jatamansi* Jones [102].

### 5.2. Anti-Cancer Effect

Current pharmacotherapy has critical tools to speed the development of new target therapies which will accelerate the final goal in the fight against cancer. Various compounds separated from *V. jatamansi* and *V. officinalis* roots were active for a variety of cellular cancer lines, both in vitro and in vivo assays. For instance, among the isolated constituents from *V. jatamansi*, the derivative IVHD-valtrate, is one of the most promising molecules that was tested against human ovarian cancer cells (A2780 and OVCAR-3), in vitro and in vivo. This compound showed inhibition of the growth and proliferation in a concentration-dependent manner. This compound also revealed low cytotoxicity to immortalized non-tumorigenic human ovarian surface epithelial cells (IOSE-144), which is very important for further research. The authors even refer that Preclinical results pointed out *IVHD-valtrate* as a potential therapeutic drug for this type of cancer [103].

Two years late, the same group evaluated jatamanvaltrates P-Y, nardostachin, and ten new valepotriates, but this time, the compounds were isolated from the whole plants and found cytotoxic activity against PC-3M cells [104]. They also examined the structure-activity relationship of these valepotriates and the results reveal a crucial 10-chlorine in the oxirane ring and the bond C3–C4. The compound valtrate was able to avoid migration of human breast cancer cells and induce apoptosis, both in vitro and in vivo. In another experiment, they discover that valtrals (A, B, and C), which are by-products of valepotriates resulting from a degradation reaction during the separation methodology from the ethanol extract prepared with the entire plant, showed selective cytotoxicity against colon cancer (HCT-8) cell lines and metastatic prostate cancer (PC-3M) [104]. Other tests performed with three new minor valepotriate isomers, jatamanvaltrates, all of them isolated from the entire plants of *V. jatamansi,* evidenced moderate cytotoxicity in the two cell lines cited above and also hepatoma (Bel7402) [105]. Jatamanvaltrate P, an iridoid ester, can inhibit the proliferation and growth, in a concentration-dependent manner, of MDA-MB-231, MDA-MB-453, and MDA-MB-468 (this last three lines corresponding to the triple-negative breast cancer) and MCF-7 cell lines. As the author’s highlight, this molecule exhibited an antitumor effect in MDA-MB-231 xenografts without noticeable toxicity, suggesting that it could be used in research for a future potential therapeutic drug against breast cancer [105]. A specific fraction obtained from *V. jatamansi* (no data provided about the parts of the plant) significantly inhibited the growth of breast cancer cells in a concentration-dependent manner by inducing apoptosis [106]. Among its therapeutic effects on insomnia and seizures the valeric acid, which is another active compound in valerian, has been reported to improve immunity against cancer [107]. Promising data also involve other compounds, in this case, fatty acids naturally occurring in seed oils such as conjugated linolenic acids (CLNs) which are likewise present in *C. ruber* and *V. officinalis)* [108].

### 5.3. Anti-Inflammatory Effect

Cravotto, et al. [109] summarized the available scientific information on the intense, in-progress investigation for novel plants, extracts, and compounds with intense anti-inflammatory activity and found out that *V. officinalis* has, among many others understudy, this potential too. Other species like *V. jatamansi* Jones also presented this bioactivity [70]. There is also a report on the anti-inflammatory activity, of a methanolic crude extract prepared from *V. wallichii* leaves (topical formulation cream) using an in vitro and in vivo screening. The authors presented data of a considerable in vitro anti-inflammatory activity obtained with the ethyl acetate fraction [110].

### 5.4. Antioxidant Effect

In natural products, different compounds are under screening for their antioxidant activity and the possible use for therapeutic strategies, for instance in degenerative diseases. Among the various therapeutic properties attributed to *Valeriana* spp., most of them correlated to valepotriates, the antioxidant properties were also investigated. According to Sudati et al. [111], *V. officinalis* extracts show a protective effect on lipid peroxidation (LPO) caused by different pro-oxidant reactive with neuro damage relevance. Dugaheh et al., [112] also studied the antioxidant effect of root extracts from *V. officinalis, N. jatamansi,* and *V. sisymbriifolia*. The best DPPH inhibition effect was obtained with *V. officinalis* extracts, but all of the tested plants inhibited beta-carotene oxidation. 

Another antioxidant activity investigation carried out with the aerial parts and roots of *V. jatamansi*, collected in pre-flowering, flowering, and post-flowering phenological stages, pointed to higher results for the first one, which could be correlated to the maximum concentration of phenolics and flavonoids. These methanolic extracts include catechin, and various phenolic acids as gallic, chlorogenic, hydroxybenzoic, caffeic, and *p*-coumaric, which largely varied among the different phenological stages, and along the altitudes. It was also highlighted that the pre-flowering stage is the most suitable for harvesting the roots containing the maximum phytochemicals amounts and antioxidant activity even for the samples which have few phenolics as the ones collected at high altitudes [113].

### 5.5. Cardiovascular Effect

Cardiovascular diseases are an important cause of death worldwide. Much research has been conducted to treat and delay these pathologies; however, much more needs to be done.

According to a report, lowering of blood pressure and heart rate, antiarrhythmic, regulation of blood lipid levels, and anti-myocardial ischemia-reperfusion injury are bioeffects that can be attributed to *Valeriana* spp. them. The vasorelaxant effect in endothelium-denuded rings was obtained with a hexane extract from *V. edulis.* The authors speculate that this effect could be related to the presence of valepotriates obtained from the hexane extract rhizomes [11]. It was suggested by Gan et al. [114] that some plant extracts, compared with the “model group”, could decrease the percentage of infarct volume, improve neurological activity, accentuate the expression of *VEGFR2* and number of new blood vessels in the cortex infarction around, given a possible further use to relive the acute cerebral ischemia-reperfusion injury [114]. 

## 6. Phytochemical Configuration of Valerianaceae

Different species under the sub-family Valerianaceae exhibits pharmacologic activities, especially in the central nervous system. These plants are known sources of different phytochemicals such as flavonoids, lignans, neolignans, and terpenoids [24,53,99,115]. Table 2 shows the various extraction methods and biological activities of compounds isolated from selected species under the sub-family Valerianaceae.

**Figure 1:**
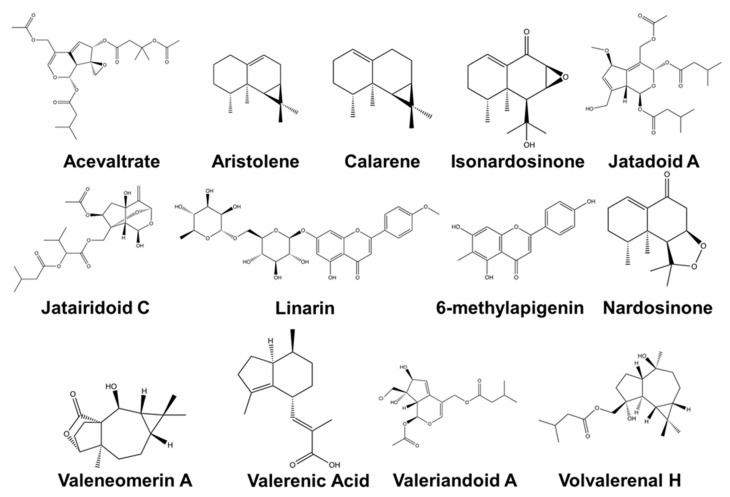
Chemical structure of selected compounds isolated from plants under the sub-family Valerianaceae with potent biological activity in the central nervous system. The chemical structures were generated using the PerkinElmer ChemDraw Prime Software Version 20.0.0.38.

Phytochemical compounds from Valerianaceae have great potential as drugs for neurodegenerative diseases (Figure 1). The compounds (–)-(8R)-neonardochinone A, (+)-(8S) neonardochinone A, and nardochinins A–D isolated from *N. jatamansi* exhibit anti-AD activity using the humanized *Caenorhabditis Elegans* AD pathological model [115]. Lignans and iridoids isolated from *V. amurensis* showed neuroprotective activity against Aβ-induced toxicity in PC12 cells [35,99,119]. AD is associated with the production and deposition of the β-amyloid peptide (Aβ) in the brain [23]. Several compounds isolated from *V. jatamansi* such as jatadoids A and B, jatairidoids A and B, valeriandoids A–C, chlorovaltrate, valerilactones A and B, and bakkenolide-H have been studied to have neuroprotective properties in MPP+-induced Parkinson’s disease model in vitro [43,44,105,127]. Sesquiterpenoids from *V. officinalis* showed AChE inhibitory activity in vitro [53,131]. AChE inhibitors are clinically used to treat neuropsychiatric symptoms of AD, PD, dementia, and schizophrenia. These compounds are promising lead compounds for discovering drugs for AD and PD.

The other compounds from Valerianaceae are also known for their relaxant effects. Aristolen-1(10)-en-9-ol, calarene, β-maaliene, and valerena-4,7(11)-diene isolated from *N. jatamansi* showed significant sedative effects in mice studies [28,75,76,116]. Treatment with valerena-4,7(11)-diene prolonged the continuous sleep time of pentobarbital-treated mice by about 2.7 times [116]. Aristolen-1(10)-en-9-ol also exhibited a sedative effect comparable to that of diazepam and this sedative property is mediated through the GABAergic system [76]. Acetoxyvalerenic acid, valerenic acid, and linarin from *V. officinalis* also showed sedative and sleep-enhancing properties in animal studies [25,128]. 2S(−)-hesperidin, 6-methylapigenin isolated from *V. jatamansi* also showed sedative properties while valtrate exhibited anxiolytic effects and significantly reduced the corticosterone level in the rat serum [24,124,126].

The compounds isolated from plants under sub-family Valerianaceae are also known for their neuroprotective properties. Valeneomerin from *V. officinalis* showed neuroprotective properties against oxidative stress [130]. Different sesquiterpenes isolated from *N. jatamansi* (isonardosinone, kanshone B, E, J, K, L and M, and nardosinone), *P. scabiosifolia* (caryophyllene oxide and *V. amurensis* (kissoone B) prevented neuroinflammation in LPS-stimulated BV2 and primary microglial cells [34,36,83,118]. Neuroinflammation is associated with multiple neurodegenerative diseases such as AD, multiple sclerosis, and PD [133]. Isopatrinioside, valeriananoid F, and structural analogs of chlorovaltrate isolated from *V. jatamansi* exhibited neuroprotective properties against CoCl_2_-induced neuronal cell death in PC12 cell [105,123]. These compounds may be further developed to prevent chemical hypoxia-induced neurotoxicity.

Some compounds are also studied for their anticonvulsant, antidepressant, and stress-reducing properties. Monoterpenoids from *V. glechomifolia* and sesquiterpenoids from *V. fauriei* showed antidepressant activity in mice [37,41]. Valerena-4,7(11)-diene from *N. jatamansi* reduced stress in animal studies [28]. Valepotriates from *V. jatamansi* and valtrates from *V. laurifolia* showed anticonvulsant properties in mice [122,132]. These studies show the variety of phytochemical compounds isolated from plant species under the sub-family Valerianaceae. Interestingly, several compounds from subfamily Valerianaceae were more associated with a certain biological effect in the central nervous system. Sesquiterpenoids such as kanshone, kissoone, nardosinone, valerinic acid, etc., were shown to have anti-neuroinflammatory properties and sedative effects. Flavonoids such as linarin and methylapigenin were known to have sedative effects. On the other hand, monoterpenoids and glycosides were shown to have neuroprotective properties against oxidative stress and toxicants in neural cell lines. These compounds have huge potential to be developed as preventive and therapeutic interventions for different diseases of the CNS. Most of the studies for these compounds are still currently in the preclinical phase and warrant more clinical studies in the future.

## 7. Extraction and Isolation Procedure of Major Compounds from Valerianaceae

To ensure the reliability and replicability of preclinical studies using compounds from plants under the sub-family Valerianaceae, standardization of plant collection and identification; phytochemical compounds extraction, isolation and validation are warranted. Generally, plants undergo several processes before a pure compound can be isolated. This includes collection and authentication of the plant, extraction, purification, and structure determination. All the studies that were reviewed in this manuscript have utilized the roots and the rhizomes of the plant as the main source for the crude extract and the isolation of its potent pure compound. Extraction, fractionation, and purification of the roots and rhizomes of Valerianaceae have no difference as compared to other plant extraction. Each roots and rhizomes undergo air-drying, soaking, and chromatographic fractionation and purification. This is a general method in all natural products research to extract all the possible compounds within the plant. The roots and rhizomes are typically used for medicinal purposes because it is believed that the essential oils from its roots produce the biologic activity [134,135]. However, the use of roots and rhizomes is more destructive for the plants than collecting their leaves and flowers or buds [136]. It was reported that in the North-West Himalayan region, the population diversity of *V. jatamansi* is getting low. In the North-East Himalayan region, this herb is also classified as an endangered medicinal plant [137]. This requires a rigorous conservation measure to preserve these plants for future research and medicinal use.

**Figure 2:**
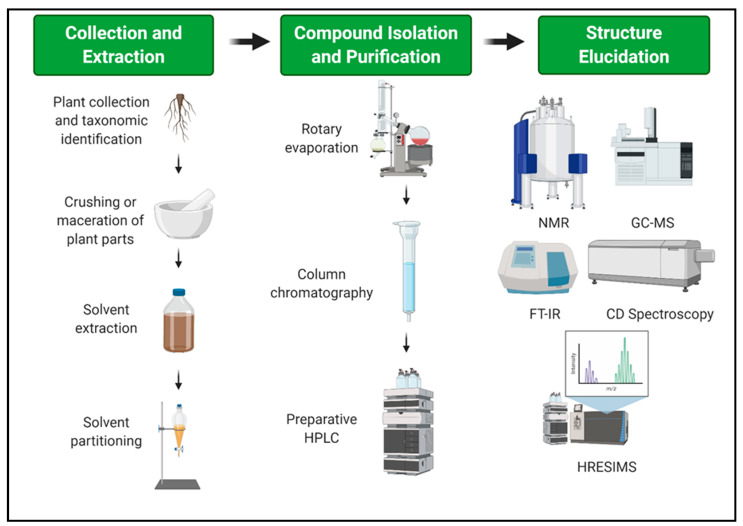
Schematic representation of the general extraction, isolation, purification, and structure elucidation of biologically active compounds from plant species under the sub-family Valerianaceae. This figure was created with BioRender.com.

For the extraction process of biologically active compounds from plants under the sub-family Valerianaceae, Figure 2 shows the schematic diagram. The roots and the rhizomes were usually air-dried at room temperature. Some of the air-dried samples were further macerated and pulverized for greater absorption of the solvent. Air-dried plant samples were extracted with ethanol, methanol, hexane, and water solvent and underwent solvent partitioning with a range of nonpolar to polar solvents such as ether, ethyl acetate, and butanol. Most authors initially extracted the pulverized sample using an alcohol solvent such as ethanol or methanol because these are the most polar among the non-polar solvents and can extract several compounds [138]. Moreover, solvents such as water, methanol, butanol, ethyl acetate, and ether were used for solvent partitioning. This is because the compounds extracted have different polarity and solubility and through this range of solvents can a researcher identify the most active compound. The most common compound isolated belongs to the group of terpenoids. Most terpenoids are non-polar and volatile, thus, solvent partitioning involving this range of non-polar to polar solvents is important to isolate and purify the potent compound [134].

Each crude extract underwent a purification process through different chromatographic techniques such as the use of silica gel column chromatography, thin-layer chromatography (TLC), and preparative high-performance liquid chromatography (HPLC). TLC is commonly done among natural products research. It is the simplest and cheapest technique to separate several components in an extract and verify the identity and purity of the compound. Furthermore, TLC serves as a guide in setting the parameters for column chromatography as a means for preparative separation [139]. Meanwhile, column chromatography is widely used as an initial separation step for the phytochemical components because of its simplicity, high capacity, and low cost. In column chromatography, the mobile phase carries the bioactive components as they pass through the stationary phase that separates the components depending on their affinity [140]. HPLC, on the other hand, is an analytic technique used to separate, quantify, and identify inorganic and organic solutes. This technique is robust and versatile that requires high pressure to elute the analyte to the detector [141]. In isolating iridoids, it was noted that these three common techniques of purification: preparative TLC, silica gel CC, and preparative HPLC were used. It is a traditional way and more feasible way to isolate compounds before undergoing more advance chromatographic techniques.

As the solute is purified, the compound is identified through its structure and molecular weight. Some of the techniques used to determine the structure of the pure compound responsible for its biological activity are nuclear magnetic resonance (NMR), gas chromatography-mass spectrophotometry (GC-MS), high-resolution electrospray ionization mass spectrophotometry (HRESIMS), Fourier transform infrared spectroscopy (FT-IR), and circular dichroism (CD). Each one has its advantage over the other but they help each other in understanding more about the discovered compound. NMR is used to give an idea about the physical, chemical, and biological property of the compound through the identification of carbon present in the compound while MS is used to identify, quantify, elucidate the structure, and determine the molecular weight of the unknown compound [142]. Mass spectrometry is an analytical tool that can give qualitative and quantitative data about the analyte and it has several types. One of these is HRESIMS which is a robust technique that can analyze the minute volume of samples that are non-volatile and thermally stable compounds. It is used when conventional techniques cannot analyze the given sample while GC-MS is a combination of two techniques making it a powerful tool in the analysis of a certain compound [143]. Gas chromatography separates the individual components while mass spectrometry characterizes the components. On the other hand, FTIR is a tool to identify the functional groups and the structure of the molecule in a given extract [142]. Finally, the CD is absorption spectroscopy that uses circularly polarized light to determine the chirality of a given compound [144]. Therefore, there is no single process in doing natural product research. The process always depends on the characteristic of the compound to be discovered.

## 8. Preclinical and Clinical Effectiveness in Humans and Patents

Although valerian has traditionally been used as a mild sedative, research results are still controversial today regarding the role of the different active compounds, the herbal preparations, and the dosage used [24,145]. In fact, in vivo studies, showed that valerian can be used as an anti-depressant [92,146] (Table 3). In particular, as abovementioned the neurobiological mechanisms of the different bioactive compounds in Valerianaceae species can be due to the effects on GABA, serotoninergic, dopaminergic, noradrenergic, and adenosine A1 receptors [86,95,147,148]. *V. glechomifolia* extract containing 96% of valepotriates (10 mg/kg) showed anxiolytic and sedative properties reducing exploratory and behavior locomotion during open field exploration without affecting memory test. Otherwise the dose of 3 mg/kg *V. glechomifolia* selectively influenced the recognition memory without effects on other behavioral parameters [149]. The anti-depressant activity of *V. glechomifolia* in mice also appears to be due to the interaction with noradrenergic and dopaminergic neurotransmission. The extract of *V. glechomifolia* can enhance its antidepressant effects such as imipramine, desipramine, and bupropion without involving the neurotransmission of serotonin and activating the noradrenergic and dopaminergic systems [149]. Holzamann et al. [58] reported the general depressive activity in rats Wistar treated with 50–150 mg/kg p.o. of the extract of *V. prionophylla* Standl., used in Mesoamerican traditional medicine for treating sleep disorders. This effect seems to be related to the capacity of *V. prionophylla* to increase pentobarbital-induced sleep time and to decrease sleep latency [150].

The anxiolytic, sedative, and memory effects of extracts from Valerianaceae species may be due to the ability to interact with the GABA_A_ receptor, possibly at the level of the subtypes (sub-unit β3; GABRB3) of receptors that mediate the effects of benzodiazepines, so producing the hypnotic and sedative activities. Administration of *V. officinalis* extracts at different dosages in BALB/c mice is related to changes in the levels of the GABRB3 protein. In particular, the extract induced an increase in the protein expression in comparison to the group of animals, which were given diazepam [155]. The anxiolytic effects have also been attributed to *V. jatamansi;* these effects may be due to the modulation of the levels of 5-HT, norepinephrine, dopamine, γ-aminobutyric acid, which by adjusting the axis hypothalamus-pituitary-adrenal axis employing β-endorphins and corticosterone [146].

The sedative and anticonvulsant effects of *V. edulis* and several valerian extracts are often related to high dosages and the different phytochemical compounds such as valerenic acids and flavonoids, present in the different Valerian species. In vivo experimental models confirmed that valerenic acid and valerian extracts have shown sedative effects [151,156,157,158]. In particular, Benke et al. [152] recently demonstrated a precise binding site on GABA_A_ receptors that showed a high affinity for valerenol and valerenic acid. Several previous studies demonstrated that the properties of valerian are related not only by the interaction with GABAA receptors but also by the involvement of adenosine receptors [148,159,160].

Several authors reported that the administration of valepotriates or *V. officinalis* root extracts in zebrafish larvae induced the modulation of c-fos and of Npas4a, involved in regulating of the neural circuits [154,161,162,163]. The modulation of these genes, induced by valepotriates, together with their ability to bind GABA_A_ receptors and histone deacetylase (HDAC) inhibitors confirm the neuroprotective effects of valerian extracts [164]. In addition to their anxiolytic and depressive effects, the extracts from *V. officinalis* can protect the neurons of the hippocampus from ischemic damage and restore behavioral deterioration. These protective effects are due to the ability of *V. officinalis* and its main constituents such as valerenic acid and acetylvalerenolic acid, to inhibit the activity of the nuclear factor (NF)-κB in vitro [165]. Other studies showed that the CNS effects of *V. officinalis, V. jatamansi,* and *N. jatamansi* can certainly also attributed to the inhibitory activity exerted on AChE [166].

Rahman and collaborators showed that the administration of an *N. jatamansi* ethanol extract to young and aged mice for 8 days enhanced memory and learning and determined a reversion of the amnesia induced by diazepam and scopolamine [153]. Among the Valerianaceae sub-family, few species have been clinically evaluated for their biological activities on CNS, despite the large traditional use, to treat insomnia, anxiety, epilepsy, and neurodegenerative diseases. The majority of controlled clinical trials available in the scientific literature are on the efficacy of improving sleep disorders of different valerian extracts. A fact sheet from the NIH Office of Dietary Supplements updated on March 2013 [167] is reported five rated randomized, controlled trials (Table 4).

The studies showed that *V. officinalis* root improved sleep quality [168], reduced sleep latency and improved the subjective sleep rating [169], decreased insomnia symptoms [170] and also improved sleep quality with results comparable to those obtained by the administration of 10 mg of oxazepam but with fewer side effects [171]. Conversely, subsequent randomized double-blind studies based on sleep parameters evaluated objectively with polysomnographic techniques detected no substantial differences on any of the measurements in comparison to the placebo group except for only one parameter [172]. The NHI document addressed to health professionals, concluded that qualitative results suggest that *V. officinalis* would be a promising strategy for insomnia and sleep-associated disorders subjective improvement. However, not all studies have produced positive outcomes and the real effectiveness of *V. officinalis* could not be proved by objective or quantitative measurements due to methodological limitations observed in most clinical studies, such as the small sample sizes, the unstandardized sources of valerian, the low rate of reproducibility of the results, etc.

Recently, Shinjyo et al. [173], based on a systematic review of 60 studies and meta-analyses, updated and re-evaluated the most reliable literature data to assess the effectiveness of *V. officinalis* in ameliorating sleep and sleep-associated disorders, yet controversial and not fully conclusive. The authors reported that the inconsistent and conflicting results of the clinical trials are maybe due to the quality and to the differences in herbal preparation in addition to the aforementioned methodological limits verified.

Among the 40 articles analyzed to assess the effectiveness of *V. officinalis* to treat sleep disorders, 30 addressed its efficacy in ameliorating sleep quality and sleep-associated problems. The additional analysis of seven articles also revealed the efficacy of *V. officinalis* in inducing positive effects on anxiety states in different stress conditions, also confirmed by another recent study on its anxiolytic activity in patients during dental surgery [174].

Other positive effects are reported in reducing the symptoms of obsessive-compulsive disorder and in preventing cognitive dysfunction rather than in reducing menopausal and postmenopausal hot flashes in women. Concluding, the results of this study suggested that the whole root rather than different extracts of *V. officinalis* is a valid and safe alternative for the treatment of sleep problems and anxiety. *V. wallichii* and *V. edulis* rich only in valepotriates and lacking in valerenic acids demonstrated sleep-promoting properties but the numbers of the studies are insufficient. The therapeutic effects on sleep were found with the use of doses of 450–1410 mg of the whole root per day for up to 8 weeks. These positive biological activities could be ensured by the standardization and characterization of the phytochemical profile of the diverse active compounds present in extracts and could be improved by the combination with different sleep-promoting herbs [173]. *N. grandiflora* is another species tested in a preliminary clinical study; the principle compound jatamansone obtained from the rhizomes significantly reduced aggressiveness and restlessness in hyperkinetic children [153].

Most common commercial Valerian preparations are generally quite safe for short-term use (for 4–6 weeks), 600 mg of valerian did not cause clinically significant effects on reaction time, alertness, and concentration the morning after ingestion [167]. No literature data is available that demonstrated the safety of long term use. In addition, little is known about their use in the woman during pregnancy and nursing and in children younger than 3 years old. However, the few side effects reported in the literature include agitation, restlessness, insomnia, headache, dizziness, itching and gastrointestinal upset. Particular attention must be paid to the potential interactions with sedative substances such as barbiturates, benzodiazepine, melatonin, alcohol, some herbs and dietary supplements with sedative properties (*Hypericum perforatum* L., *Piper methysticum* G. Forst) due to the potential additive effects. Cytotoxicity was detected only in vitro for valepotriates with no carcinogenic effects in animal models. These compounds are not always present in commercial valerian preparations. There is report that the roots and rhizomes of *Valeriana officinalis* have been used in official medicine in the form of various dosage for more than 240 years [175]. Moreover, Valeriana drugs in Russian medicine are used in mono form, which increases the importance of this plant for the treatment of a wide range of diseases [175]. Despite the wide use of these herbs in traditional medicine as well in the official medicine, more advanced clinical studies are still necessary on other plant species of this subfamily to establish the numerous biological activities associated with their pharmacological applications.

## 9. Conclusions

The studies reported and discussed in the present review article have indicated the multifaceted biological activities of many species from the Valerianaceae sub-family. Reviewed studies indicated that species from the genus *Valeriana*, particularly species like *V. officinalis*, *V. jatamansi, V. fauriei, V. amurensis, V. glechomifolia,* and *V. polystachya* have positive effects to treat disorders related to the Central Nervous System dysfunctions, such as insomnia, anxiety, and epilepsy. The mode of action of valerian compounds is mainly associated with the modulation of GABAergic transmission. Nevertheless, validated clinical studies are still necessary to establish the numerous and interesting biological activities associated with the pharmacological applications of the species.

## Figures and Tables

**Table 1:** Selected studies reporting the effects of Valerianaceae species on the central nervous system.

Plant Species	Extract/Compound	Effect/Main Findings	Model/Assays	Dose	Administration	Reference
*Nardostachys chinensis* Batalin (synonym of *Nardostachys jatamansi* (D.Don) DC.)	Valerena-4,7(11)-diene from roots	Anti-stress, inhibited stress-induced excitatory behaviors and reduced stress-induced blood corticosterone, cerebral serotonin, and dopamine levels	Mice, a model of acute stress (restraint stress for 15 min)	300 µg/cage	Inhalation	[28]
*Nardostachys chinensis* Batalin (synonym of *Nardostachys jatamansi* (D.Don) DC.)	Sesquiterpenoids isolated from underground parts	Alleviate the Alzheimer’s disease-like symptom of paralysis in worms	*Caenorhabditis elegans* Alzheimer’s disease pathological model	50 μM	Added to the culture medium	[29]
*Nardostachys jatamansi* (D. Don) DC.	Rhizomes 70% ethanol extract	Anti-stress effect, inhibited cold restraint stress-induced oxidative stress	Rats, the cold restraint stress model	200 and 500 mg/kg	Orally	[30]
*Nardostachys jatamansi* (D. Don) DC.	Root fractions	Anti-inflammatory effects reduced lipopolysaccharide-induced inflammatory response	Lipopolysaccharide-induced inflammation in murine peritoneal macrophages and mice model of lipopolysaccharide-induced endotoxin shock	10–100 μg/mL	Intraperitoneally	[31]
*Nardostachys jatamansi* (D. Don) DC.	Root 70% ethanol extract	Anxiolytic effects	Mice, models of anxiety (elevated plus maze, open field test, light-dark box test, and Vogel’s conflict test)	250 mg/kg	Orally	[32]
*Nardostachys jatamansi* (D. Don) DC.	Sesquiterpeniods	Anti-neuroinflammatory effects	BV2 microglial cells	Several (10–80 μM)	In vitro	[33,34]
*Valeriana amurensis* P. Smir. ex Kim.	Isolated compounds from roots and rhizomes	Ameliorate amyloid-beta-induced cognitive dysfunction	Amyloid-beta1-42 induced Alzheimer’s disease mice model	0.2–0.8 g/kg	Intrahippocampal injection	[35]
*Valeriana amurensis* P. Smir. ex Kim.	Petroleum ether, ethyl acetate, *n*-butanol, and aqueous extract, and kissoone B from roots and rhizomes	Anti-inflammatory and neuroprotective effects	Cell (THP-1 cells as surrogates for microglia, SH-SY5Ycells as surrogates for neurons, and U373 cells as surrogates for astrocytes) and mice models	400 μM kissoone B and 100 μg/mL extracts	Intragastric	[36]
*Valeriana fauriei* Briq.	Sesquiterpenes from the roots	Antidepressant	Mice, Forced swim test	20 mg/kg, during seven consecutive days	Orally	[37]
*Valeriana fauriei* Briq.	Commercial root extract	Reduction the incidence of prenatal stress related-psychiatric disorders	Rats, prenatal stress model, evaluation of behavioral patterns and changes in protein levels in the prefrontal cortex	100 mg/kg/day, administered on postnatal days 35–56	Orally	[38]
*Valeriana fauriei* Briq.	Aqueous extract	Antinociceptive effect	Mice, a model of fibromyalgia (induced by intermittent cold stress)	100 mg/kg/day for 24 days	Orally	[39]
*Valeriana glechomifolia* Meyer	Diene valepotriates fraction from underground parts	Antidepressant, interaction with dopaminergic and noradrenergic neurotransmission	Mice, tail suspension test (TST), and forced swimming test (FST)	0.25–20 mg/kg	Orally	[40]
*Valeriana glechomifolia* Meyer	Valepotriate-enriched extract from aerial and underground parts	Antidepressant potential, prevent lipopolysaccharide-induced sickness and depressive behavior	Mice submitted to a forced swimming session as a stressful stimulus (experimental model of depression associated with inflammation)	10 mg/kg	Orally	[41]
*Valeriana glechomifolia* Meyer	Valepotriate-enriched fraction from aerial and underground parts	Anti-inflammatory activity, inhibition of leukocytes migration	Formalin test in CF1 mice and Wistar rat’s leukocytes migration assay	1, 10 and 30 mg/kg; 0.1–1 g/mL	Orally	[42]
*Valeriana jatamansi* Jones	Bakkenolides from rosts	Neuroprotective effects	1-Methyl-4-phenylpyridinium-induced neuronal cell death in human dopaminergic neuroblastoma SH-SY5Y cells	1.5, 5 and 15 μM	In vitro	[43]
*Valeriana jatamansi* Jones	Iridoids from roots	Neuroprotective effects	1-Methyl-4-phenylpyridinium-induced neuronal cell death in human dopaminergic neuroblastoma SH-SY5Y cells	3, 10 and 30 μM	In vitro	[44]
*Valeriana jatamansi* Jones	Root ethanol extract	Anxiolytic action	Mice, elevated plus maze, light/dark box test, and spontaneous activity	1.2, 2.4 and 4.8 g/kg, for 10 days	Orally	[45]
*Valeriana jatamansi* Jones	Root and rhizome (Zhi zhu xiang) 35% ethanol extract	Anti-anxiety activity	Empty bottle stimulated rats, open field test, and the elevated plus-maze test	1.2 g/kg, for 7 days	Orally	[46]
*Valeriana jatamansi* Jones	Iridoid-rich fraction from roots and rhizomes	Antidepressive	Unpredictable mild stress mouse model	5.73, 11.47 and 22.94 mg/kg	Orally	[47]
*Valeriana officinalis* L.	Extracts from roots	Anxiolytic and antidepressant effect	Mice elevated plus maze test and forced swimming test	100–1000 mg/kg	Orally and intraperitoneally	[21]
*Valeriana officinalis* L.	Root ethanol extract and valerenic acid	Anxiolytic effects, reduction in anxious behavior	Rats, elevated plus maze	3 mL/kg extract and 3 mg/kg valerenic acid	Intraperitoneally	[23]
*Valeriana officinalis* L.	Roots aqueous extract	Anticonvulsant effects	Mice, pentylenetetrazole-induced clonic seizure	0.25, 0.5 and 1 g/kg	Intraperitoneally	[48]
*Valeriana officinalis* L.	Root extract and valerenic acid	Memory function, cell proliferation, neuroblast differentiation, serum corticosterone, and lipid peroxidation	Mice, D-galactose-induced aging model	100 mg/kg extracts and 340 μg/kg valerenic acid	Orally	[49]
*Valeriana officinalis* L.	Root ethanol extract	Antinociceptive effects, pain modulation	Mice, Tail-Flick Test, Acetic Acid Writhing Test	50, 200 and 800 mg/kg	Intraperitoneally	[50]
*Valeriana officinalis* L.	Root extract	Anti-stress effects	Mice, exposure to physical stress psychological in a communication box	100 mg/kg/0.5 mL	Orally	[22]
*Valeriana officinalis* L.	Root aqueous and ethanol extracts	Anticonvulsant effects	Zebrafish (*Danio rerio*), an animal model used to study clonic-like behaviors	1 mg/mL; 5 mg/mL	Dissolved in aquarium water	[51]
*Valeriana officinalis* L.	Root ethanol extract	Protective effects against ischemic injury in the hippocampal pyramidal neurons	Gerbils subjected to ischemia/reperfusion injury	100 mg/kg	Orally	[52]
*Valeriana officinalis* var. *latiofolia*	Sesquiterpenes and a monoterpenoid from roots	Inhibition of acetylcholinesterase	In vitro and in vivo in mice	0.65, 1.30 and 2.6 mg/kg/day, for 90 days	Intragastric	[53]
*Valeriana officinalis* L.	Root aqueous extract	Elucidation of mechanisms of neuroprotective action against rotenone-induced cellular damage	Theoretical analysis (microarray data)	-	-	[54]
*Valeriana officinalis* L.	Eighteen root compounds	Inhibition of GABA aminotransferase	Molecular docking and molecular dynamics simulations	-	-	[55]
*Valeriana officinalis* L.	Root aqueous extract	Protective action against rotenone effects (counteract Cortical spreading depression propagation velocity and C6 glioma cytotoxicity)	Cortical spreading depression (in vivo) and C6 glioma cell culture (in vitro) models	250 mg/kg/day, for 15 days	Orally	[56]
*Valeriana polystachya* Smith	Extract from roots and rhizome, and isolated compounds from roots and rhizomes	Inhibition of acetylcholinesterase and prolyl oligopeptidase activities	In vitro	200 μg/mL extract and 150 μM of isolated compounds	In vitro	[57]
*Valeriana prionophylla* Standl.	Roots and rhizomes 50% ethanol extract	Anxiolytic, antidepressant, and hypno-sedative effects	Swiss mice and male Wistar rats, open field, rota rod, elevated plus-maze, forced swimming, strychnine- and pentobarbital-induced sleeping time, pentylenetetrazole-induced seizures, and the inhibitory avoidance tests	50, 100 and 150 mg/kg	Orally and intraperitoneally	[58]
*Valeriana wallichii* DC (synonym of *Valeriana jatamansi* Jones)	Roots and rhizomes dichloromethane extract	Antidepressant effect	Mice, acute toxicity, studies forced swim test, locomotor activity and measurement of biogenic amines	10, 20 and 40 mg/kg	Orally	[59]
*Valeriana wallichii* DC (synonym of *Valeriana jatamansi* Jones)	Roots and rhizomes essential oil	Antidepressant effect	Mice, acute toxicity, studies forced swim test, locomotor activity, measurement of biogenic amines and effect of nitric oxide modulators	10, 20 and 40 mg/kg	Orally	[60]
*Valeriana wallichii* DC (synonym of *Valeriana jatamansi* Jones)	Root aqueous extract	Sleep quality improvement	Rats, estimation of the sleep-wake profile, electroencephalogram delta activity, and estimation of regional brain monoamines.	200 and 300 mg/kg	Orally	[61]
*Valeriana wallichii* DC (synonym of *Valeriana jatamansi* Jones)	Rhizome methanol extract	Neuroprotective effect	Mice, 1-methyl-4-phenyl-1,2,3,6-tet-rahydropyridine-induced Parkinson’s disease model	50, 100 and 200 mg/kg	Orally	[62]

**Table 2:** Extraction methods and biological activities of compounds isolated from selected species under the sub-family Valerianaceae.

Major Compound	Known Biological Activity	Isolation Techniques Used *	Detection Methods **	First Author and Year
***Nardostachys jatamansi* (D.Don) DC.**				
(–)-(8R)-neonardochinoneA and (+)-(8S)neonardochinoneA	Anti-Alzheimer’s disease (AD) activity	Silica gel CC, MCI gel CC, and Sephadex LH-20 CC	HRESIMS NMR XRC	[115]
Nardochinins A-D		Silica gel CC, MCI gel CC, and Sephadex LH-20 CC	HRESIMS NMR XRC	[29]
Kanshone C—inhibits SERT and Desoxo-nachinol A—enhances SERT	Natural serotonin regulator using SERT activity assay	Silica gel CC and preparative HPLC	HRESIMS NMR XRC	[78]
Aristolen-1(10)-en-9-ol	Sedative effect via GABAergic system	Silica gel CC and preparative HPLC	GC-FID GC-MS	[76]
Valerena-4,7(11)-diene	The stress-reducing effect in mice	Silica gel CC and preparative HPLC	GC-MS	[28]
Valerena-4,7(11)-diene and b-maaliene	Sedative effects in mice	Silica gel CC, gel permeation chromatography, and HPLC	GC NMR	[116]
Aristolene, calarene, and valerena-4,7(11)-diene	Sedative effects in mice	Silica gel CC, gel permeation chromatography, and HPLC	GC/GC-MS NMR	[75]
Kanshone L, Kanshone M	Anti-inflammatory effects in BV2 and primary microglial cells	Silica gel CC and preparative HPLC	NMR HRESIMS	[34]
Sesquiterpenoids: Kanshone J and Kanshone K	Anti-inflammatory effects in BV2 and primary microglial cells	Solvent partition, CC, and HPLC	NMR HRESIMS	[34]
Nardosinone, Isonardosinone, Kanshone E, Kanshone B	Anti-inflammatory effects in BV2 and microglial cells	Silica gel CC	NMR MS	[83]
Compounds 5 and 6	Cytotoxic activity against a neuroblastoma cell line	Silica gel CC	FT-IR MS NMR	[117]
***Patrinia scabiosifolia* Link**				
Caryophyllene oxide	Anti-inflammatory activity in BV-2 cells	Distillation	GC-MS	[118]
***Valeriana amurensis* P. Smirn. ex Kom**.				
Kissoone B	Anti-inflammatory and neuroprotective effects	Percolation, Sephadex LH-20 CC, and paper chromatography	EIMS NMR	[36]
Xiecaoside E and Lignin 11-17	Neuroprotective effects in PC12 cells	Silica gel CC and preparative HPLC	FT-IR NMR	[119]
Lignans (e.g., (þ) pinoresinol-4, 4′-di-O-β-D-glucopyranoside, (þ) 8-hydroxypinoresinol-4′-Oβ-D-glucopyranoside); Iridoids (e.g., patrinoside and kanokoside A)	Activity on cerebral cholinergic function and neuroprotective effect from an αβ-induced cognitive deficit in mice	Silica gel CC, octadecyl silica gel CC, and preparative HPLC	NMR EIMS	[35]
Heishuixiecaoline A, B, and C, volvalerenal C, (+) pinoresinol-4,4′-di-O-β-D-glucopyranoside, (+) pinoresinol-8-O-β-D-glucopyranoside, and 8-hydroxypinoresinol-4,4′-di-O-β-D-glucopyranoside	Neuroprotective effects in PC12 cells	AB-8 macroporous resin CC and silica gel CC	HRESIMS NMR FT-IR	[99]
***Valeriana fauriei* Briq.**				
8α-acetoxyl-3α,4α,10-trihydroxyl-guaia-1(2)-ene-12,6α-olide and 2-Ethylhexyl-4-hydroxybenzoate	An antidepressant activity using forced swim test in a mouse model	Silica gel CC and Sephadex LH-20 CC	FT-IR MS NMR	[37]
***Valeriana glechomifolia* F.G. Mey.**				
Valtrate, Acevaltrate, 1-β-acevaltrate, 1-β-aceacevaltrate and isovaltrate	Activity on depressive-like behavior in mice	Supercritical CO_2_ (SCCO_2_) extraction and HPLC	HPLC	[41]
Valtrate, acevaltrate, and 1-β-acevaltrate	Inhibition of Na^+^/K^+^-ATPase activity in the brain hemispheres of rat	Ultrasonic bath and preparative TLC	NMR	[120]
***Valeriana jatamansi* Jones**				
Rupesin E	Anticancer and pro-apoptotic activity against glioma stem cells.	Silica gel CC, Sephadex LH-20 CC, and semi-preparative HPLC	NMR	[121]
(4β,8β)-8-methoxy-3-methoxy-10-methylene-2,9-dioxatricyclo[4.3.1.0]decan-4-ol and (1S,3R,5R,7S,8R,9S)-3,8-epoxy-1- O-ethyl-5-hydroxyvalechlorine	Neuroprotective effects in PC12 cells	Silica gel CC, semipreparative HPLC, Sephadex LH-20 CC, preparative TLC	NMR ECD FT-IR UV Vis HRESIMS	[105]
Valepotriate	The anti-epileptic effect in mice	Silica gel CC	NMR HPLC	[122]
Isopatrinioside and Valeriananoid F	Neuroprotective effects in PC12 cells	Silica gel CC, Sephadex LH-20 CC, preparative TLC, and preparative HPLC	NMR HRESIMS FT-IR MCP UV-Vis	[123]
Valtrate	The anxiolytic effect in rats	Chromatography in AB-8 macroporous adsorption resin, silica gel CC, and TLC	NMR EIMS FT-IR UV Vis	[124]
Iridoids: Jatadoids A and B	Neuroprotective effects in SH-SY5Y cells	Silica gel CC, TLC, ODS CC, and preparative HPLC	NMR HRESIMS	[44]
Jatairidoids A–C	Neuroprotective effects in SH-SY5Y cells	Silica gel CC, ODS CC, and preparative HPLC	NMR HRESIMS EIMS FT-IR	[125]
Valeriandoids A–C and chlorovaltrate	Neuroprotective effects in SH-SY5Y cells	Silica gel CC, ODS CC, and preparative HPLC	NMR HRESIMS EIMS FT-IR	[44]
2S(−)-hesperidin	Sedative and sleep-enhancing properties in mice	Silica gel CC	UV-Vis NMR EIMS	[24]
6-methylapigenin	Anxiolytic and sleep-enhancing properties in mice	Silica gel CC and C18 column chromatography	UV-Vis NMR EIMS	[126]
Valerilactones A and B, and bakkenolide-H	Neuroprotective effects in human dopaminergic neuroblastoma SH-SY5Y cells	Silica gel CC, ODS CC, and preparative HPLC	NMR HRESIMS ESIMS FT-IR	[127]
***Valeriana officinalis* L.**				
Acetoxyvalerenic acid and valerinic acid	Sleep promoting properties in mice model	Soxhlet extraction, rotary vacuum evaporation, and C18 CC	HPLC UV-Vis	[128]
Valerenic acid	GABA_A_ receptor modulator using a larval zebrafish seizure model	ASE^®^ 200 solvent extraction system	HPLC	[129]
Volvalerenal H, Volvalerenal I, Volvalerenal J, Volvalerenal acid K, and Densispicnins C	Acetylcholinesterase inhibitory activity	Silica gel CC, Sephadex LH-20 CC, preparative TLC, and preparative HPLC	NMR HRESIMS FT-IR	[53]
Valeneomerin A, Valeneomerin B, Valeneomerin D	Neuroprotective effects against H2O2 induced oxidative stress in SH-SY5Y cells	Silica gel CC, RP-MPLC, and Sephadex LH-20 (MeOH) CC, and preparative TLC	NMR HRESIMS FT-IR XRC	[130]
Linarin	Sedative and sleep-enhancing property	Silica gel CC	NMR EIMS UV-Vis	[25]
Volvalerenals A-E and volvalerenic acids A-C	Weak acetylcholinesterase inhibitory activities	Silica gel CC, and Sephadex LH-20 CC	NMR HRESIMS EIMS XRC	[131]
***Valeriana laurifolia* Kunth**				
Valtrate acetoxyhydrine, valtrate, isovaleroyloxyhydrine, and valtrate chlorohydrine	Anticonvulsant property in mice	Silica gel CC, and preparative TLC	NMR HRESIMS EIMS FT-IR	[132]

* CC—column chromatography, HPLC—high-performance liquid chromatography, ODS—octadecyl silica; TLC—thin layer chromatography. ** ECD—Electronic circular dichroism; EIMS—Electrospray ionization mass spectrometry; FT-IR—Fourier-transform infrared spectroscopy; GC-FID—Gas chromatography with flame-ionization detection; HRESIMS—High-resolution electrospray ionization mass spectrometry; MCP—Modular circular polarimeters; MS—Mass spectroscopy; NMR—nuclear magnetic resonance; UV Vis—Ultraviolet-visible spectroscopy; XRC—Xray crystallography.

**Table 3:** In vivo studies on the effects of some species belonging to the Valerianaceae sub-family on the Central Nervous System.

Compound/Species	Animal Model	Dosage	Outcomes	Ref.
Valerenic acid derivatives	Male mice (c57Bl/6N)	3 mg/kg	Anxiolytic effect	[151]
Valerenic acid derivatives	Mutant mice (GABA_A_ receptor b3 subunit mutation)	From 1 to 30 mg/kg	Anxiolitic effects	[152]
*V. glechomifolia*	Swiss male CF1 mice	1, 3, and 10 mg/kg	Sedative effects	[149]
*V. prionophylla*	Swiss female mice; Wistar male rats	50, 100, and 150 mg/kg	Anxiolytic; antidepressant; hypno-sedative effects	[58]
*N. chinensis*	Different animal model	Different dosages	Antidepressant; anticonvulsant; neuroprotective and antiparkinson activities; cognition and memory improvement	[153]
*V. jatamansi*	Kunming mice	Ethanolic extract	Anxiolytic effects; No drug dependence	[146]
*V. officinalis*	Zebrafish larvae	0.3 g/kg, 0.9 g/kg	Regulation of neural-activity genes	[154]
*V. officinalis*	BALB/c mice	1, 2.5, 5, and 7 mg/mL	Modulate GABAA subunit β3 receptors; sedative effects	[155]
*V. edulis*	Wistar male rats	2.5 mg/10 g; 5 mg/10 g	Anticonvulsant properties	[156]

**Table 4:** Systematic reviews reporting the effectiveness of some species of Valerianaceae sub-family on CNS.

Species	Systematic Review	Indication	Conclusions	Source
*V. officinalis*	5 clinical trials	Sleep disorders	Not sufficient for determining the effectiveness	[167]
*V. officinalis*	60 studies and meta-analysis	Sleep disorders	Sufficient for determining the effectiveness but standardization and quality control is needed	[173]
*N. grandiflora*	Preliminary clinical studies	Aggressiveness, restlessness, stubbornness, sleep disorders	Further studies are needed	[153]

## Data Availability

All data related to the review manuscript are presented in the manuscript in the form of tables and figures.
